# Generalization of the Dynamic Clamp Concept in Neurophysiology and Behavior

**DOI:** 10.1371/journal.pone.0040887

**Published:** 2012-07-19

**Authors:** Pablo Chamorro, Carlos Muñiz, Rafael Levi, David Arroyo, Francisco B. Rodríguez, Pablo Varona

**Affiliations:** 1 Grupo de Neurocomputación Biológica, Dpto. de Ingeniería Informática, Escuela Politécnica Superior, Universidad Autónoma de Madrid, Madrid, Spain; 2 Department of Neurobiology and Behavior, University of California Irvine, Irvine, California, United States of America; Mount Sinai School of Medicine, United States of America

## Abstract

The idea of closed-loop interaction in *in vitro* and *in vivo* electrophysiology has been successfully implemented in the dynamic clamp concept strongly impacting the research of membrane and synaptic properties of neurons. In this paper we show that this concept can be easily generalized to build other kinds of closed-loop protocols beyond (or in addition to) electrical stimulation and recording in neurophysiology and behavioral studies for neuroethology. In particular, we illustrate three different examples of goal-driven real-time closed-loop interactions with drug microinjectors, mechanical devices and video event driven stimulation. Modern activity-dependent stimulation protocols can be used to reveal dynamics (otherwise hidden under traditional stimulation techniques), achieve control of natural and pathological states, induce learning, bridge between disparate levels of analysis and for a further automation of experiments. We argue that closed-loop interaction calls for novel real time analysis, prediction and control tools and a new perspective for designing stimulus-response experiments, which can have a large impact in neuroscience research.

## Introduction

The idea of a direct closed-loop interaction with neurons goes back to the beginnings of electrophysiology in the 1940s when the work of George Marmount and Kenneth Cole resulted in the voltage clamp technique that measures currents across the membrane of excitable cells while holding the membrane voltage at a set level [1], [2]. Later on, the dynamic clamp technology for *in vitro* and *in vivo* electrophysiology [3], [4] has produced many examples of successful closed-loop interactions with neural systems. The dynamic clamp protocols build a voltage-dependent current-injection cycle to introduce artificial membrane or synaptic conductances into living neurons. It has been used to investigate a large variety of membrane properties and to create hybrid circuits of real and artificial neurons and synapses [5]–[9]. As different software implementations have become available both under Windows [7], [10]–[12] and real time Linux operating systems [13]–[17], this technique has turned into a widely used tool for studying neural systems at the cellular and circuit levels (for a review see [5], [18]–[20]).

The dynamics of neurons and neural networks can only be observed partially, i.e., through a subset of variables that reflect their current state such as intra– or extra–cellular membrane potential, calcium concentration, blood oxygen level, etc. Classic dynamic clamp only considers membrane potential for observation and current injection for stimulation. A further complication is that neural systems are highly nonlinear and adaptive, usually working in transient regime [21]–[23], which adds to the problem of partial observation. Thus, the mechanisms to extract information from them and the way to drive effective stimulation are very limited. In this context, closed-loop interaction provides a large variety of possibilities to characterize dynamics from partial measurements and to exert control or induce learning through activity-dependent stimulation.

**Figure 1:**
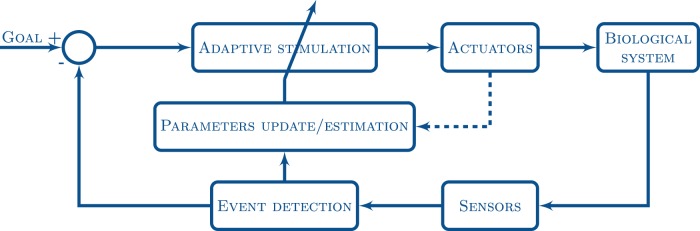
Schematic representation of the goal-driven closed-loop for the activity-dependent stimulation used in the three examples discussed in this paper. The activity of the biological system is monitored through a set of sensors (e.g. microelectrodes, cameras). A given goal drives the detection of specific events that are used to control the adaptive stimulation (through specific actuators) that will lead to this goal. Simultaneously, the output of the event detection and the stimulation can be used for identification purposes by updating or estimating the parameters that control this loop. Examples of goals for the closed-loop interaction are to exert control, reveal or characterize the dynamics, or to achieve the automation of a experiment as we illustrate in the next sections.

Given a specific goal, an adaptive closed-loop protocol can automatically search for or characterize dynamics, achieve effective control or induce learning that relies on precise or varying timing, duration, amplitude and/or location of the stimulation. In this paper we show several examples of how the dynamic clamp concept can be further generalized into a wide variety of activity dependent interactions that go beyond electrical recording and stimulation. Note that similar concepts to the voltage clamp, such as calcium clamp techniques, exist since the 80s [24], [25]. However, they not always follow a close-loop feedback approach or still pose a number of problems in the context of neurophysiological studies [26]. The examples discussed in this paper arise in different experimental contexts but they all share a common goal-driven closed-loop illustrated in Fig. 1. The activity of the biological signal is monitored through a specific set of sensors (e.g. microelectrodes or cameras) and an event detection algorithm is used to drive the adaptive stimulation protocol through the actuator (a microelectrode that conveys a current, a microinjector that delivers a neurotransmitter, a stepper motor that applies a mechanical stimulus, etc.). The output of the detection and the stimulation can be used for identification purposes by updating or estimating the parameters used in this loop. The goal-driven nature of the closed-loop is crucial as only in this case can the adaptive stimulation be evaluated and then modulated online by the update of the loop parameters.

The paper is organized as follows: First we describe a closed-loop for activity-dependent neurotransmitter or neuromodulator microinjection with the goal of controlling the spiking-bursting activity of motoneurons; in the next section we illustrate closed-loop video event driven stimulation for behavioral control in neuroethological studies; then we provide an example of activity-dependent mechanical stimulation that allows to automate the search for receptive fields throughout the sensory-motor transformation. Finally, we discuss the need for real time or online event detection algorithms and internal representations to build a new generation of adaptive activity-dependent stimulation protocols for a broad range of research in neuroscience.

## Results

### Closed-loop Drug-microinjection

In dynamic clamp protocols, microelectrodes are used to record voltage and deliver currents in an activity-dependent manner. Such closed-loop allows to build an artificial chemical synapse by modeling the dynamics of a synaptic current triggered by a presynaptic spike. This is generally done with one or several equations that readily provide the current for each voltage value [3], [4], [12].

A more realistic artificial chemical synapse can be achieved by substituting current with a controlled amount of neurotransmitter or neuromodulator injection in a given neuron or circuit. This injection can be delivered as a function of a specific event detected in an electrophysiological recording. The drug delivery, controlled by a computer that is monitoring a signal used to build a stimulus-response loop, can be executed through a microinjector. Closed-loop drug-microinjection is particularly relevant since in the most common cases the event to trigger the microinjection is the occurrence of one or several action potentials at a specific time not known a priori. The event detection monitoring in the closed-loop scheme shown in Fig. 1 provides a solution to this problem.

Here we will illustrate closed-loop drug microinjection with a specific example in the framework of the study of neural signatures of cell-specific intraburst insterspike intervals [27]–[29]. Neural signatures are robust and reproducible spike timings within the bursting activity of individual neurons. They were first described in the context of the study of central pattern generator circuits [27]. Experimental and modeling results show that neural signatures can have an important role in the activity of neural networks to identify the source of the information or to contextualize a message [30]–[32]. In order to address the functional effect of neural signatures, a procedure to change the number of action potentials and the temporal structure of the intraburst spiking activity is required. We describe bellow a simple experiment to perform this task with the heart motoneurons of the crab *Carcinus maenas* using our closed-loop system. The goal of this activity-dependent stimulation is to achieve specific number of spikes in the bursting activity of a neuron through acute chemical inhibition.

**Figure 2:**
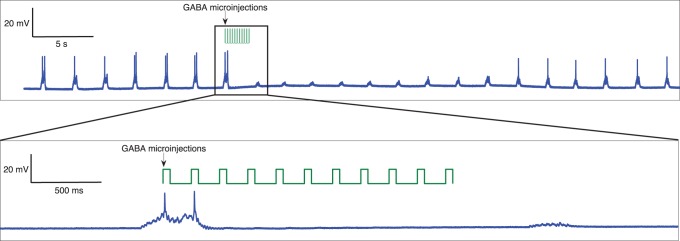
Transient effect of GABA microinjections on cardiac cells in a traditional open-loop protocol with periodic stimulation. The top panel shows the effect of GABA on the membrane potential of a CPG neuron from the cardiac ganglion of *Carcinus maenas*. The vertical arrow indicates the instant in which a burst of periodic GABA microinjections (vertical lines) of 50 ms of duration and separated by 200 ms takes place. These injections produce a transient inhibitory effect on the bursting activity. The bottom panel is a blow up of the squared region on the top panel. Single pulses evoke a much more transient response as shown in Fig. 4, which is used to control the number of spikes in each burst during the closed-loop experiment.

In an open-loop experiment the heart central pattern generator (CPG) from the cardiac ganglion of the crab *Carcinus maenas* was subjected to microinjections of gamma-aminobutyric acid (GABA). Figure 2 shows that GABA microinjections have a transient inhibitory effect on the neurons of the cardiac ganglion. The effect of this inhibition depends on the amount and timing of the microinjections and can be adjusted so that the inhibition is mild and short (enough to modify the number of spikes or the duration of single bursts). This type of stimulus was used in the closed-loop microinjection protocol that we describe below.

In the proposed closed-loop drug-microinjection, the membrane potential of one neuron is measured and an adaptive stimulation protocol of GABA microinjection is implemented by coupling the microinjections to the detection of specific events in an eletrophysiological recording. The microinjections are delivered at a desired location with a Picospritzer. The stimulation onset and duration is precisely controlled through the activity-dependent protocol.

**Figure 3:**
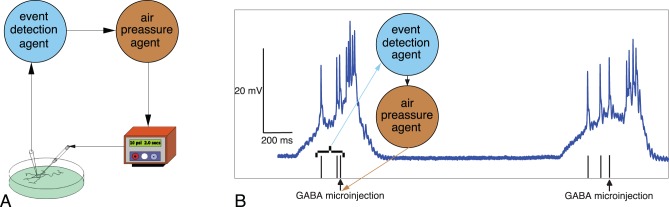
Activity-dependent drug microinjection. Panel A shows a schematic representation of the closed-loop drug stimulation protocol. In this example, the membrane potential of a neuron is monitored by an event detection algorithm to perform the activity-dependent drug microinjection. When an event is detected, the software sends a signal to the microinjector and the neurotransmitter or neuromodulator is released. Panel B shows the real time (RT) stimulation protocol we employed in the experiments discussed in this section. This adaptive protocol consists of a double 1 mM GABA injection (two 40 ms pulses separated by 30 ms) when the third spike is detected at the beginning of a burst of a cardiac neuron (vertical lines indicate the detection of single spikes, arrows indicate the instant in which the microinjection takes place). The resulting inhibitory closed-loop is used to achieve a desired number of spikes in the bursting activity of these neurons.

Figure 3 illustrates the details of this activity-dependent closed loop. The membrane potential of a cardiac cell is monitored by the real time (RT) software which runs an event detection algorithm to perform the activity-dependent drug microinjection. When an event is detected, the software sends a signal to the microinjector and GABA is released. The right panel shows the RT stimulation protocol we employed in the experiments. This protocol consists of a double 1 mM GABA injection (two 40 ms pulses separated by 30 ms) when the third spike is detected at the beginning of a burst of a cardiac neuron. In fact, any protocol based on sequential event detections involving different temporal and spatial scales can be implemented to build the loop.

**Figure 4:**
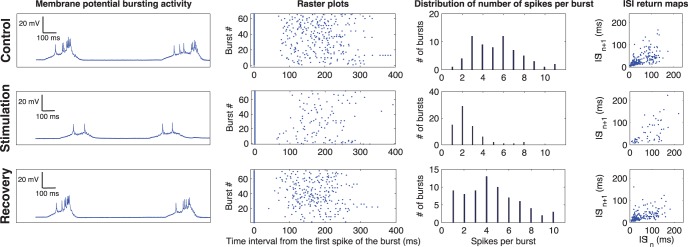
Results of the activity-dependent drug stimulation protocol. The rows on the first column show the membrane potential time series during control (top row), stimulation (middle row) and recovery after washout (bottom row). The rows on the second column show the raster plots for control (top), stimulation (middle) and recovery (bottom). The rows on the third column show the distribution of the number of spikes in each burst for the three time series. Finally, the panels on the fourth column show the inter-spike intervals (ISI) return maps during control (top), stimulation (middle) and recovery (bottom). Note that during the stimulation, the number of spikes per burst drastically decreased because of the RT activity-dependent GABA microinjections.

The effect of the activity-dependent GABA microinjection protocol evoked by the real time detection of three action potentials in a CPG neuron is shown in Fig. 4. For the characterization of the activity during the control, the inhibitory closed-loop protocol and the recovery period, we used the raster plots of the spiking activity, the distribution of the number of spikes per burst and the inter-spike interval return maps. The top row shows the control activity (irregular bursts with a large variability in the number of spikes in this preparation). The middle row shows the activity during the closed-loop stimulation period. The activity-dependent stimulation protocol was able to regularize the bursting activity and maintain it within a given number of spikes per bursts without periodic injections of GABA. At the beginning of this adaptive protocol there were successive microinjections that automatically became less and less frequent leading to the activity shown in the middle panel of the first column of Fig. 4. The bottom row shows the activity after the stimulation protocol was stopped and the neurons went back to their normal bursting regime.

We have illustrated a simple protocol of real time event-driven drug microinjection to change neural signatures and achieve a desired state in the spiking-bursting activity of CPG neurons. This novel type of activity-dependent chemical interaction can be applied to the study of many aspects of neuromodulation and neurotransmitter stimulation, and to achieve control of natural or pathological states through a temporally precise drug release. The protocol can be further enhanced through the monitoring of signals from different neurons or nerves and multiple drug delivery at different sites. Note that these families of protocols may use stimuli that depend not only on instantaneous measurements, but also on the previous (adequately long) history of the recordings.

### Closed-loop Video-event Driven Stimulation

Following the same strategy illustrated in the previous section, animal behavior can be monitored and stimuli can be driven as a function of events that evolve in time and are not periodic or predictable a priori. This is especially relevant in those studies where the focus is on behavioral activity that is triggered by the interaction of the animal with its own environment. It is also important for conditional learning tasks that heavily rely on the animal’s previous activity to decide what stimulus comes next. While offline video analysis is widespread in behavioral studies [33]–[35], online video tracking and particularly video event-driven stimulation remains quite unexplored in neuroscience research. Online video tracking is often limited for automation of observations [36], with very few exceptions mainly in the context of maze studies in rodents [37]. In this section we show an example of online video tracking and device triggered in a closed-loop to implement neuroethological activity-dependent stimulation protocols.

Video event driven stimulation can be used to build model-driven conditional training experiments, learning protocols and behavioral control procedures. Behavioral monitoring can be implemented through online video tracking while stimulation is driven through the online control of visual, auditory, olfactory, mechanical or electrical cues. There is a wide variety of possibilities to monitor animal behavior and deliver activity-dependent stimulus by building actuator control signals which can have an adaptive temporal structure based on events detected from the online video tracking and/or other behavioral sensors. It is also possible to define events from multiple modalities when available and to combine different stimulation techniques.

**Figure 5:**
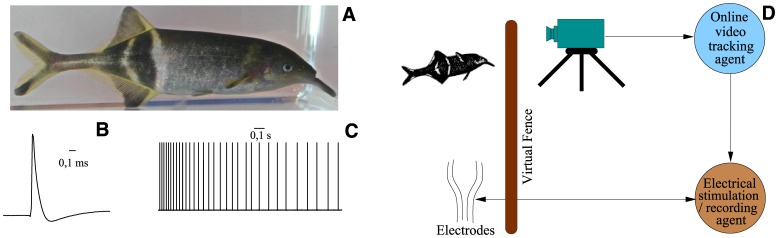
Closed-loop video-event driven stimulation. A: Electric fish *Gnathonemus petersii*. B: Single electrical organ discharge of this fish. C: a typical train of activity (signals are squared in this plot). D: Schematic representation of the closed-loop video-event driven stimulation.

We illustrate the use of these protocols with an example of activity-dependent stimulation for the elephant fish *Gnathonemus petersii* (see Fig. 5). This fish has poor eyesight and uses a weak electric field to find food and to navigate [38]–[40]. *Gnathonemus petersii* is also a well-known animal model for the study of electric communication [41], and its signaling has also been used to assess water quality [42]. In our example we use adaptive electrical stimulation as a function of the fish position detected from online video-tracking to build a virtual fence. Panel D in Figure 5 illustrates the setup for the online video tracking and the activity-dependent stimulation protocol.

In many cases, fast online tracking of the position of the animal in a controlled environment can be achieved in a simple manner by subtracting consecutive frames from the camera recording. This can be easily implemented with, for example, opencv libraries (http://opencv.willowgarage.com). Thus, events for the triggering of the stimulus in our example can come from the camera that monitors the location of the fish, and/or from the electrical activity of the animal. This activity is recorded in real time from cables immersed in the water tank and the corresponding signals are amplified, acquired by a DAQ board and processed by a computer (see Fig. 5D). The electrical stimulus is generated in the computer (conditioned in amplitude and frequency to make it aversive but not harmful to the fish), sent to the DAQ board and delivered by the immersed cables (the actuators in the general close-loop scheme shown in Fig. 1).

**Figure 6:**
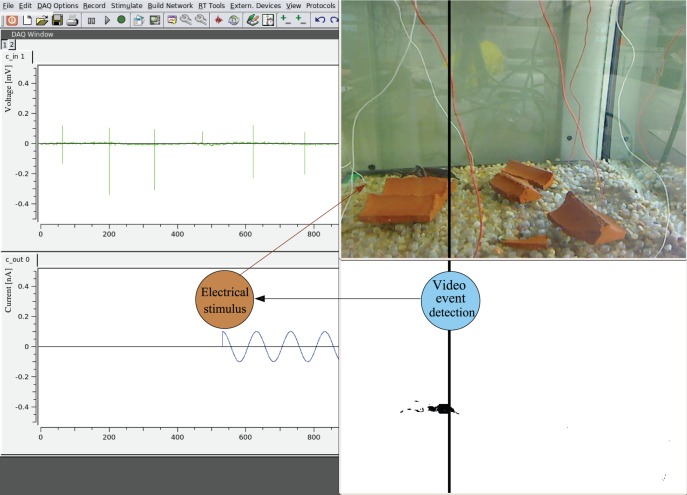
Virtual water fence through position-dependent stimulation. When the fish crosses a virtual barrier (vertical black line on the right panels) an aversive stimulus is delivered (bottom left) so that the fish stays in a specific space of the water tank. The top left panel shows the electrical activity of the fish.

Since the goal in this example of closed-loop video-event driven stimulation is to build a virtual fence for the fish, we used a sinusoidal aversive electrical stimulus that was delivered as a function of the fish position in the tank. Right panels in Figure 6 depict the virtual fence as a vertical black line both in the online video panel and in the tracking panel (below). This figure also illustrates the fish electrical activity (left top panel) and the aversive signal delivered when the fish crosses the virtual fence (below).

**Figure 7:**
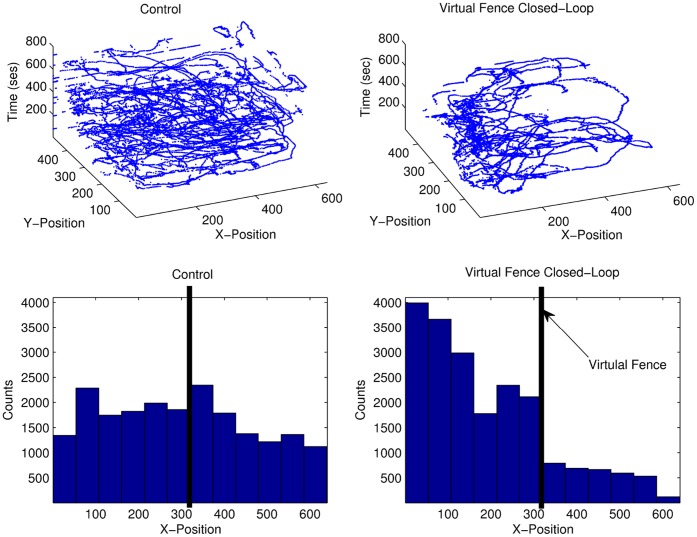
Analysis of the fish position tracking. In the control experiment, the fish explored the tank without a preferred position (left panels). Once the virtual fence closed-loop stimulation started, the fish stayed mainly on the left part of the tank where no stimulation was received (right panels). Note the abrupt change in the histogram at pixel 300 in the horizontal axis of the camera, which corresponds to the position of the virtual fence.

Figure 7 shows the result of the analysis of the tracking of the fish during a control experiment with no stimulation (left panels) and during the virtual fence activity-dependent stimulation protocol (right panels). Both experiments lasted 800 seconds. In the control experiment, the fish explored the tank without a preferred position (left panels). In the closed-loop system, the position of the fish was monitored and the aversive stimulus was delivered when the animal crossed the virtual barrier (located at pixel 300 in the horizontal axis of the camera). Once the virtual fence closed-loop stimulation started, the fish remained mainly on the left part of the tank where no stimulation was received (right panels in Fig. 7). The protocol can be adapted so that the stimulus gets increasingly stronger as the fish gets closer and closer to a virtual fence. Similarly, the protocol can also include the online analysis of the electric fish signals as well as its position to deliver the stimulus. All these families of protocols can be used to train the fish to be in a specific region of the water tank without having a physical barrier and to study its signaling in different behavioral contexts.

### Closed-loop Mechanical Stimulation

In a last example, we illustrate a further generalization of the activity-dependent closed-loop in electrophysiology, in this case oriented to automatically find receptive fields throughout the sensory-motor transformation with an adaptive mechanical stimulation. Mechanical stimulation is widely used to study sensory encoding and sensory-motor transformation (e.g. see [43]–[46]). Realistic mechanical stimulation is needed in these studies as the correct sensory input will evoke a natural response at any stage of the nervous system [47]. Novel types of mechanical stimulation protocols that include artificial closed-loops between different stages of the sensory-motor transformation can unveil the underlying dynamics of information processing. The stimulation of mechano-receptor neurons is achieved through a large variety of devices that generate movement [48]–[51]. In particular, a stepper motor can act as a precise mechanical stimulator since, as an electric device that divides a full rotation into a large number of steps, it can be turned to a very accurate angle. Speed and acceleration can also be controlled by sending the appropriate commands at precise time windows, which in some cases requires dedicated hardware, programmable logic or the use or real time software technology [47].

To illustrate a closed-loop control of a stepper motor for mechano-sensory stimulation we will use an *in vitro* preparation of the mollusk *Clione limacina*. A combination of simplicity, accessibility of the system and variability of behaviors make this animal especially attractive for a complete understanding of the sensorimotor transformation [52], [53]. *Clione* is a planktonic mollusk that swims by rhythmic movements of a pair of wings and the tail. During swimming *Clione* maintains a head up orientation [54]–[56], under the control of signals from a pair of gravimetric organs, the statocysts [56]–[58]. Each statocyst contains a stone-like structure, the statolith, that moves inside the sphere under the influence of gravity. The statolith excites the sensory neurons that line the internal wall of the statocyst. The statocyst activity has a strong influence on the wing and tail motor systems that control the body orientation [52]. A deviation from the vertical orientation of the animal evokes compensatory changes in wing and tail motions. In addition, the statocysts have been shown to play a major role in generating *Clione*’s hunting behavior [53], [59]–[61], which consists in a series of fast loops in varying planes to scan the surrounding space in search of prey [62]. After removal of one statocyst, *Clione* can maintain orientation, although it is slightly off the vertical plane [58]. We used this fact and manipulated only one of the statocyst to produce a motor response.

**Figure 8:**
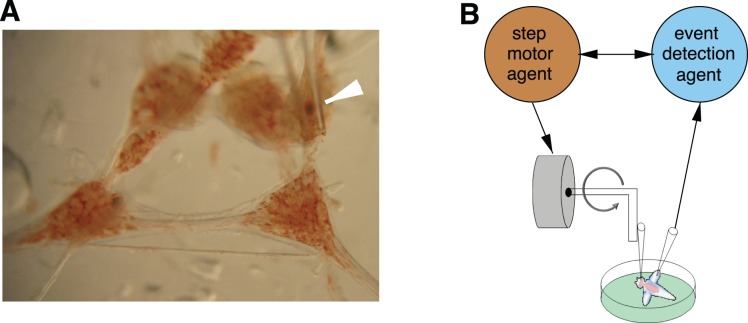
Experimental setup for the closed-loop mechanical stimulation. (A) Close up of the preparation showing *Clione*’s nervous system with the pipette holding the statocyst (white arrow). (B) Schematic representation of the activity-dependent mechanical stimulation closed-loop. The figure depicts the suction pipette that holds the gravimetric organ and the recording electrodes used to detect events that drive the motor movements.

To build a realistic method of statocyst stimulation, we detached the statocyst from the pedal ganglion while leaving the nerve to the cerebral ganglion intact. The statocyst was gently sucked into a glass pipette whose tip diameter corresponded to that of the statocyst (see Fig. 8). The pipette was attached to an arm connected to the stepper-motor. By closely adjusting the length of the pipette to align its tip with the axis of rotation of the arm, we were able to move the statocyst in one plane of a particular orientation, either posterior/anterior or left/right. Different stimulation paradigms involving speed, acceleration and directional changes can be tested with this setup, in which only the statocyst moves while the rest of the nervous system remains static. The motor controller has to send a sequence of commands distributed in accurate time intervals to the motor inputs. Since our goal is to implement an activity-dependent control of the rotation, we have used the analog inputs of a data acquisition board to record neural activity and its digital output to send commands to the motor (for details on the real time control of stepper-motors see [47]). To build the activity-dependent stimulation, extracellular recordings from the wing nerve were done with a steel electrode as described in [53], [62].

**Figure 9:**
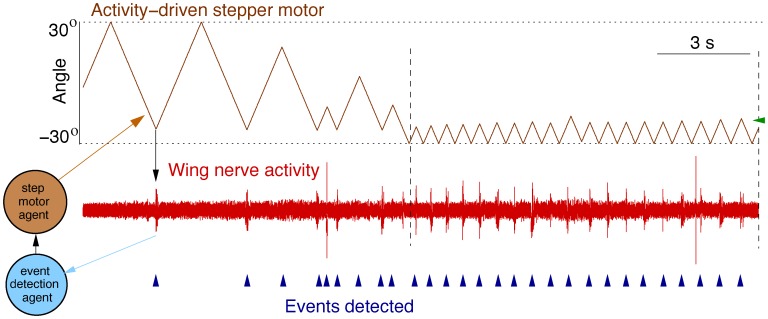
Automatic receptive field search through the stepper motor activity-dependent stimulation. The figure shows the simultaneous recording of the stepper motor movement (top row), the wing motor nerve (middle row) and the real time burst detection on the wing nerve (bottom row). The motor is automatically sweeping through a range of angles. The software monitors the occurrence of stereotyped bursts in the activity of the wing nerve. After a burst detection or when a defined maximum angle is reached (horizontal dotted lines), the motor changes direction. Vertical dashed lines indicate a region where a strong response of wing motoneurons was observed in response to a stimulation around −21° (green horizontal arrow).

Figure 9 shows the results of the closed-loop used in this example with the goal of automatically finding receptive fields of motoneurons reacting to the mechanical stimulation of the gravimetric organ. The following protocol was used: The motor sweeps through a range of angles (top row in Fig. 9). The software monitors the occurrence of a stereotyped burst in the activity of the wing nerve as recorded by the extracellular electrodes (middle row in this figure, the burst events are indicated by blue vertical arrows at the bottom row). After a burst detection, the motor changes direction. When another burst is detected or a maximum angle is reached (horizontal dotted lines), the motion turns back in the opposite direction. Vertical dashed lines in the middle row in Figure 9 point out a region found with this closed-loop protocol where a strong response to the motor movement was observed for an angle of around −21° (green horizontal arrow). This receptive field was automatically detected by the activity-dependent stimulation protocol.

The above experiment is just an illustrative example of the use of closed-loop mechanical stimulation to achieve an automatic search for activity. The same protocol can be used throughout the sensorimotor transformation to find receptive fields at the sensory, central nervous system or motor system stages. In fact, a wide variety of adaptive protocols involving complex spatial or temporal relations between the detected events and the mechanical stimuli can be implemented using the same approach. In this method (as well as in the other examples above), as a pure software-based solution based on the scheme depicted in Fig. 1, all efforts are directed at relatively simple programing using experimental equipment that is already present in any laboratory without the need for additional expensive hardware. Thus, the real time detection of events and the control of actuators are compactly integrated, which allows a researcher to define and modify a large variety of detection algorithms and control parameters.

## Discussion

In this paper we have shown three representative examples of how the dynamic clamp concept can be generalized to build novel activity-dependent protocols to exert control, drive behavior or reveal dynamics both in neurophysiological and behavioral experiments. Traditional dynamic-clamp experiments monitor voltage activity and instantaneously drive a corresponding electrical current to implement artificial membrane conductances or synapses. The examples presented illustrate that other types of monitoring and stimulation mechanisms are possible for a wide range of applications in neuroscience research by following and expanding the philosophy used in dynamic-clamp protocols with the adaptive closed-loop approach represented in Fig. 1. In particular, we have first described an activity-dependent drug-microinjection to precisely deliver GABA as a function of specific events detected in membrane potential activity. This inhibitory closed-loop produces regularized activity with the desired number of spikes as a result of a reduced number of microinjections whose timing is determined by the ongoing activity. Then we have illustrated an online video-event driven stimulation to control the behavior of an electric fish. In this protocol the position of the fish was tracked online and an aversive electrical signal was used to build a virtual fence. Finally in the last section, the mechanical stimulation of a gravimetric organ was triggered by the ongoing activity of a nerve to automatically find receptive fields of neurons reacting to this stimulation. All these protocols were implemented following the goal-driven closed-loop illustrated in Fig. 1. The goal given to each experiment was used to evaluate and modulate the adaptive stimulation, in particular its timing, duration, and/or amplitude.

From a theoretical point of view, building new types of closed-loops for neuroscience research requires to enlarge the techniques and procedures presented in the previous sections, which used relatively simple event-detection and stimulation protocols. The research and development of new interaction closed-loops for neuroscience and neuroethology call for novel online event detection, characterization of the dynamics, and stimulus exploration tools. As we have discussed above, stimuli can be constructed not only in response to instantaneous activity but also by integrating past measurements. Moreover, we have highlighted the convenience of using all possible sources of information (i.e., to integrate multimodality both in the recording and the stimulation) to construct the actuation law. Novel types of activity-dependent stimulation protocols need methods to automatically select the proper stimulus to modify given dynamics. The aim of these procedures is not only to achieve a desired dynamical behavior, but also to unveil the inner characteristics of the system(s) by means of the analysis of events detected in correspondence to realistic stimuli. Therefore, methods and tools are required to simultaneously address identification, representation and exploration tasks for closed-loop interactions.

Online event detection is one of the most critical components of the closed-loop technology, since the controllability of closed-loop schemes requires the extraction of the significant parts of time series in a short span of time. There is a real need to design and implement accurate and fast methods to handle non-stationarity. Procedures based on asymptotic behavior of time series are not suitable in this context, since in many cases the core of neural activity is determined by transient dynamics and non-stationary processes [22]. As alternatives we can consider model based and time-frequency methods, along with the symbolic analysis of time series. Transient dynamics can be reproduced by a proper dynamical system model [23], [63] and, consequently, event identification can be defined as a procedure to determine some parameters of the underlying dynamical system model [64]. The interleaving of event detection and internal representation is very time consuming and dependent on the selected model, which makes this strategy not advisable in some cases. A possible solution can be provided by time-frequency methods, since they can be successfully applied to dynamics characterization [65], [66]. Another option for approaching transient dynamics is to deal with coarse-grained versions of the associated time series and resort to the framework of applied symbolic theory. An efficient and accurate way to translate time series into symbolic representations is drawn by their ordinal patterns [67], which have been successfully applied to detect determinism [68], to the estimation of dynamical parameters [69], and to control chaotic systems [70]. Finally, on-line detection of events can be only performed if statistics are computed for short time series, and thus sliding windows must be used in order to meet this need. Entropy estimation for short time series can be further improved by using, for example, the Lempel-Ziv complexity measure [71].

It is important to emphasize that modern types of activity-dependent stimulation can act simultaneously in different time and spatial scales in order to bridge between different levels of analysis and deal with intrinsic limited observation and stimulation capabilities. Multimodality both in the recording and the stimulation can also lead to improved results for the tasks of revealing dynamics and achieving control. The goal-driven closed-loop interaction scheme depicted in Fig. 1 allows the processing of multiple sensors and actuators.

We can identify two main factors that may impede the rapid progress of the generalization of closed-loop activity-dependent stimulation in neuroscience: The cost of commercial hardware and software solutions, and the inertia to go beyond the classical ways of thinking about stimulus-response experiments in neuroscience. We hope that the examples that we have provided in this paper and the fast development of non-commercial software can contribute in this direction. In this context, we are building RTbiomanager [16], a tool to take advantage of real time technology to build activity driven protocols in a wide variety of experiments. All examples shown in this paper were implemented using this software.

Beyond the examples illustrated here, multiple electrode and modern optical techniques (voltage and calcium imaging, optogenetics, two-photon microscopes, fMRI setups, laser stimulation) can largely benefit from the generalization of the dynamic-clamp concept that we have discussed in this paper. Cell cultures and stem-cell research could also use a large variety of adaptive activity-dependent protocols for targeted differentiation purposes.

Extracting information from the nervous system from partial measurements and limited stimulation methodologies is an extremely difficult task. In this context, novel goal-driven closed-loop interactions will lead to automate experiments, to reveal dynamics otherwise hidden under traditional stimulus-response protocols, and to achieve faster and better control on natural or pathological dynamics.

## Methods

Although the activity-dependent stimulation protocols were described in the main text and illustrated in the figures, in this section we provide further details on the methods for the three different experiments discussed in this manuscript.

### Closed-loop Drug-microinjection

Adult male and female shore crabs were used for the illustration of the closed-loop drug-microinjection. The heart was accessed by removing the overlying carapace. Once extracted, it was pinned ventral side up in a silicone elastomer (Sylgard) petri dish. The lateral walls were cut out and the heart ganglion was dissected out from the surrounding muscles. The isolated cardiac ganglion was bathed by *Carcinus maenas* saline (in mM: 433 NaCl, 12 KCl 12 CaCl_2_2H_2_O, 20 MgCl_2_6H_2_O, 10 HEPES, adjusted to pH 7.60 with 4 M NaOH). Membrane potential was recorded from the anterior motor neuron LC3 using 3M KCL-filled microelectrodes (10–20 Mohms). The signal was amplified on a A-M Systems neuroprobe amplifier (model 1700) and acquired by a National Instruments PCI-MIO-16E-4 card. GABA was dissolved in *Carcinus maenas* saline to a final concentration of 1 mM. This solution was directly applied onto the LC3 soma using a Picospritzer III microinjector (Parker Hannifin Corp.).

### Closed-loop Video-event Driven Stimulation

Large specimens of *Gnathonemus petersii* were acquired from a local aquarium. Silver cables located in the corners of the water tank (c.f. Figs. 5D) were used to deliver the aversive stimulus and to record the electrical activity of the fish. We used a Logitech C905 USB camera to monitor the location of the fish in real time. The RTBiomanager software implemented the online video-tracking to instantaneously calculate the position of the fish and to deliver the aversive stimulus via a DAQ National Instruments PCI-6251 acquisition board, which was also used to record the electrical activity of the fish.

### Closed-loop Mechanical Stimulation

*Clione* limacina specimens were collected at St. John’s, Newfoundland, and sent to our lab in Madrid. The preparation, including cerebral, pedal, and abdominal ganglia with the tail and wing nerves, was pinned to a Sylgard lined petri dish as previously described [53], [62]. Extracellular recordings were made by stainless steel electrodes. The details of the real time control of the stepper motor can be found in [47]. The stimulation and the recording were performed using a National Instruments PCI-MIO-16-E4 acquisition board. A stand-alone code to control stepper motors under Linux with RTAI (RealTime Application Interface) is available in our website http://www.ii.uam.es/~gnb/rtmotor.
